# Safety and Efficacy of Single Anastomosis Sleeve Ileal (SASI) Bypass Surgery on Obese Patients with Type II Diabetes Mellitus during a One-Year Follow-up Period: A Single Center Cohort Study

**DOI:** 10.34172/aim.2023.55

**Published:** 2023-07-01

**Authors:** Seyed Morteza Mousavi Naeini, Mir Mohsen Toghraee, Nasser Malekpour Alamdari

**Affiliations:** ^1^Baqiyatallah University of Medical Sciences, Tehran, Iran; ^2^Department of General Surgery, Modarres Hospital, Shahid Beheshti University of Medical Sciences, Tehran, Iran; ^3^Critical Care Quality Improvement Research Center, Shahid Modarres Hospital, Shahid Beheshti University of Medical Sciences, Tehran, Iran

**Keywords:** Bariatric surgery, Diabetes mellitus, Metabolic surgery, Morbid obesity, Single anastomosis sleeve Ileal (SASI) bypass

## Abstract

**Background:**

We aimed to evaluate the safety and efficacy of single anastomosis sleeve ileal (SASI) bypass surgery on obese patients with type II diabetes mellitus during a one-year follow-up period.

**Methods:**

We included patients with a body mass index (BMI) more than 35 kg/m^2^ and at least one-year history of type II diabetes mellitus. We excluded patients aged under 25 or above 66 years, those who were not candidates for surgery, needed another bariatric surgery, and those not willing to participate in the study. All the patients were visited in the outpatient office on the 10^th^ and 45^th^ days as well as the 3^rd^ month of the post-operative period until the end of the first year.

**Results:**

in this study, we investigated 14 male (23.0%) and 47 female (77.0%) morbidly obese patients with type II diabetes mellitus who underwent SASI bypass. The mean excess weight loss (%EWL) was 60.99±15.69 and the mean total weight loss (%TWL) was 30.39±6.52 at the end of the one-year follow up. Finally, 44 patients (72.1%) had a complete and 15 patients (24.6%) had partial remission of type II diabetes mellitus. Of note, severe complications were recorded in two patients (3.2%). Paired *t* test analysis demonstrated a significant decrease for fasting plasma sugar (FBS) after one-year follow-up in comparison with FBS before surgery (*P*<0.0001). Furthermore, this difference was observed in HbA1c (*P*<0.0001).

**Conclusion:**

SASI bypass is an effective method for weight loss and controlling type II diabetes mellitus.

## Introduction

 As a complex multifactorial disease, obesity is one of the emerging world-wide healthcare concerns. The worldwide prevalence rates of overweight and obesity have increased to double since 1980. Nearly one third of the world’s population is currently classified as overweight or obese.^1^ Obesity is defined as abnormal or excessive fat accumulation that may consequently impair health, and many studies suggest that without any intervention, the resolution of obesity is uncommon.^2^ A recent meta-analysis showed that both obesity and morbid obesity (body mass index [BMI] > 25 kg/m^2^) increase the mortality due to COVID-19 infection.^3^

 Diabetes is known as a major independent risk factor of cardiovascular diseases. The pathophysiology of type II diabetes is related to insufficient number of pancreatic islet beta-cells which have the responsibility to regulate chronic energy excess, and this leads into insulin resistance, glycemic load, and obesity.^4^

 Bariatric surgery has been reported to have superiorities over medical treatments for managing morbid obesity.^5^ The mechanism of weight loss is the determining factor for classification of bariatric procedures. Bariatric surgery has resulted in satisfactory outcomes by application of malabsorptive, restrictive, or mixed weight loss mechanisms.^6^ Delivery of undigested food to distal small bowel releases some mediators that affect weight loss and improve glucose hemostasis.^7^

 Long-term data on sleeve gastrectomy with transit bipartition (SG-TB) has been recently released by Santoro et al. SG-TB is performed similar to duodenal switch (DS); however, there is no complete exclusion of the duodenum to minimize nutritional alterations.^8^ A technical modification has been applied to one anastomosis duodenal switch and one anastomosis gastric bypass by Mahdy et al by adding a single anastomosis between the gastric pouch and the ileum, known as single anastomosis sleeve ileal (SASI) bypass.^9^ Type II diabetes mellitus and gastroesophageal reflux disease (GERD) were controlled more efficiently by SASI bypass in comparison with SG. In this regard, both procedures had similar weight loss 6 months postoperatively as well as comparable complication rates.^10^ The SASI bypass has the metabolic advantage of sleeve gastrectomy and gastric bypass with further evaluations of the GI system feasible using standard endoscopes.^11^

 This novel procedure, that can control type II diabetes mellitus and weight loss, is the basis of our study. The current research aimed to study both the safety and efficacy of SASI bypass surgery on obese patients with type II diabetes mellitus during a one-year follow-up period.

## Materials and Methods

 This experimental study was conducted on morbidly obese patients with type II diabetes mellitus in the bariatric surgery clinic of Nikan hospital, Tehran, Iran, between May 2017 and May 2018. A total of 61 morbidly obese patients with type II diabetes mellitus patients were enrolled by nonprobability sampling. The protocol of the present study was registered at the ethics committee of our institutional review board. We included patients with a BMI more than 35 kg/m^2^,at least one-year history of type II diabetes mellitus, HbA1c > 6%, and (simultaneously) FPG > 100. We excluded those patients aged under 25 or above 66 years, those who were not candidates for surgery, needed revisional bariatric surgery, and those not willing to participate in the study. Some other suitable bariatric procedures were offered to the patients who refused to participate in the study. All the patients were informed of the study process, the technique of the surgery (which is adherent to IFSO (International Federation for the Surgery of Obesity and Metabolic Disorders)standards) and the possible complications. Thereafter, they signed a written informed consent form and underwent the SASI procedure.

 All patients were assessed by history taking, physical examination and biochemical tests, before surgery. An endoscopy was performed for all the patients before performing the intervention. Moreover, *Helicobacter pylori* test was done and the infection was eradicated before surgery in case of positivity. Next, abdominal ultrasound was conducted to assess the liver and to rule out the presence of gallstones. Before the intervention, all the patients were evaluated by a multidisciplinary team and a number of tests, including echocardiography, polysomnography, and psychiatric evaluation were done for them. As prophylaxis against deep vein thrombosis (DVT), low molecular weight heparin was administered subcutaneously for all the patients 1 to 2 hours prior to and 2 weeks after surgery.

 We investigated the percentage of excess (%EWL) and total weight loss (%TWL) based on formulas in the study by Sewefy et al,^12^ as the primary outcomes. In addition, we evaluated the remission of type II diabetes mellitus which was defined as a fasting plasma sugar (FBS) level less than 100 mg/dL or an HbA1c level less than 6%, while the patient receives no medications. Also, a minimum reduction of 1% in the level of HbA1c or 25% in the FBS level with hypoglycemia medications, was considered as partial improvement.^13^

###  Surgery Technique

 In this operation, patients are placed in the French position in a steep reverse Trendelenburg position and the surgeon stands between the patient’s legs. General anesthesia accompanied by endotracheal intubation is used for patients. In the classic sleeve gastrectomy, the greater omentum is separated from the stomach in the beginning of the operation. The dissection is started 5 cm from the pylorus, and then will continue upwards to cut the short gastric vessels and remove every attachment from the left crus. The surgeon dissects any adhesions between the stomach and pancreas. To achieve an appropriate sleeve, a 37-French calibration tube is used as a guide. Stapling is performed at 6 cm proximal to the pylorus, by a linear cutting stapler which is continued upwards to divide the stomach. The staple line is then oversewn using a running PDS suture 2/0. After identifying the Treitz ligament, the surgeon measures the total small intestine. The intestinal loop with the middle of the intestine is then lifted to the gastric sleeve through dividing the greater omentum, and then is fixed to the sleeved stomach at the pyloric ring using a stay suture. Then, a 45 linear cutting stapler is used to perform a stapled isoperistaltic side-to-side anastomosis and the surgeon places 2.5 to 3 cm of the length of the anastomosis at the inferior side of the dissected antrum. Afterwards, a two-layer 3/0 PDS running suture is used to close the defect of the gastro-jejunal anastomosis, and the presence of leaks is assessed using the air-leak test. Early antithrombotic prophylaxis is started four hours after the surgery and clear fluids are started six hours after the surgery. After the operation, proton pump inhibitors are administrated for 3 months and DVT prophylaxis continues for 2 weeks.

###  Follow-up

 All patients were visited in the clinic on the 10^th^ and 45^th^ days as well as the 3^rd^ month of the post-operative period. Subsequently, the follow-up visits were continued every three months until the end of the first year. The patients were also visited in the office if they had developed symptoms during the time between their follow-up visits. The patients were followed for one year after the SASI bypass. Of note, all the patients continued a liquid diet for 10 days, followed by a soft diet for two weeks. Subsequently, the patients were prescribed a diet including low calories and high protein levels. Other nutrients were gradually added under supervision of a dietitian. The patients were prescribed daily multivitamin and calcium citrate to be regularly taken for 6 months and then to be gradually reduced. We prescribed Neurobion injections every 15 days in the first month and then monthly for a 6-month duration. Ursobile capsules (300 mg BD for 6 month) were prescribed for all the patients and ferrous sulfate was given to women every day.

 All patients underwent a comprehensive biochemical evaluation every three months, including complete blood count, FBS, HbA1c, liver function as well as serum albumin, iron, and vitamin D levels. Other specific tests were conducted based on the patient’s clinical condition. In addition, all the complications were recorded in a pre-designed checklist.

###  Statistical Analysis

 Data were analyzed using statistical package for social sciences (IBM Corp. Released 2013. IBM SPSS Statistics for Windows, version 22.0. Armonk, NY: IBM Corp). We used Shapiro-Wilk test for checking the normality of the data. Descriptive analysis was used to report quantitative and qualitative variables as mean ± SD. Appropriate parametric tests and their non-parametric equivalents were performed. A *P* value less than 0.05 was considered as statistically significant.

## Results

###  Pre-operation and Follow-up Data

 All patients were aged between 29 and 66 years (mean age 49.66 ± 9.35 years) with at least one-year history of type II diabetes mellitus. In this study, we analyzed 14 male (23.0%) and 47 female (77.0%) morbidly obese patients with type II diabetes mellitus who underwent SASI bypass. All the patients completed the one-year follow-up. The mean body mass index (BMI) was 43.70 ± 5.81 kg/m^2^ (Table 1).

**Table 1:** Preoperative Characteristics of Study Individuals

**Variable**	**Number**	**Minimum**	**Maximum**	**Mean±SD**
Age (y)	61	29	66	49.66 ± 9.35
Ideal weight(kg) (14)	61	46.0	82.0	55.42 ± 8.81
Pre-operative weight (kg)	61	87.0	158.0	113.70 ± 16.68
Hight (cm)	61	143	188	161.92 ± 8.45
Pre-operative BMI (kg/m^2^)	61	35.27	63.82	43.70 ± 5.81
Pre-operative FBS	61	110	284	171.28 ± 30.05
Pre-operative HbA1c	61	6.1	11.1	8.53 ± 1.21

BMI, Body mass index; FBS, Fasting blood sugar; HbA1c, hemoglobin A1c.

###  Complications 

 The technique was converted to sleeve gastrectomy in two patients, in one case due to severe diarrhea, which was not tolerable by the patient after 3 months and in another case due to severe hypoalbuminemia and weight loss after 6 months.

 Four patients (6.55%) developed short-term gastritis which manifested with epigastric pain and vomiting. Gastritis was completely improved with conservative management. Five patients (8.19%) with heartburn, none of whom had GERD prior to surgery, were managed conservatively. Two patients (3.27%) had diarrhea three months after the surgery, which was tolerable and we managed them medically. One patient (1.63%) complained of constipation, three months after surgery, which was managed medically.

###  Effect of SASI on Weight loss and Diabetes Control

 The mean %EWL was 60.99 ± 15.69 and the mean %TWL was 30.39 ± 6.52 at the end of the one-year follow-up (Table 2). Normal levels of blood glucose were achieved two months after the surgery in all the patients. Finally, 44 patients (72.1%) had complete and 15 patients (24.6%) had partial remission of type II diabetes mellitus. Nutritional supplements were tapered in all the patients after sixth months. Table 2 demonstrates the post-operation data.

**Table 2:** Postoperative Characteristics of Study Individuals

**Variable**	**Number**	**Minimum**	**Maximum**	**Mean±SD**
Weight after 1 year (kg)	59	60	108	78.98 ± 11.53
Post-operative BMI (kg/m^2^)	59	22.45	43.49	30.05 ± 4.11
%EWL	59	30.36	94.34	60.99 ± 15.69
%TWL	59	16.19	46.49	30.39 ± 6.52
One year after operation FBS	59	86	121	99.15 ± 7.68
One year after operation HbA1c	59	5.1	5.8	5.43 ± 0.18

BMI, Body mass index; EWL, %Excess weight loss; TWL, % total weight loss; FBS, Fasting blood sugar; HbA1c, hemoglobin A1c

 Paired *t *test analysis demonstrated a significant difference between pre-operative FBS and FBS levels after one year of follow-up (*P* < 0.0001). Furthermore, this difference was observed in HbA1c, as well (*P*< 0.0001)

**Figure 1:**
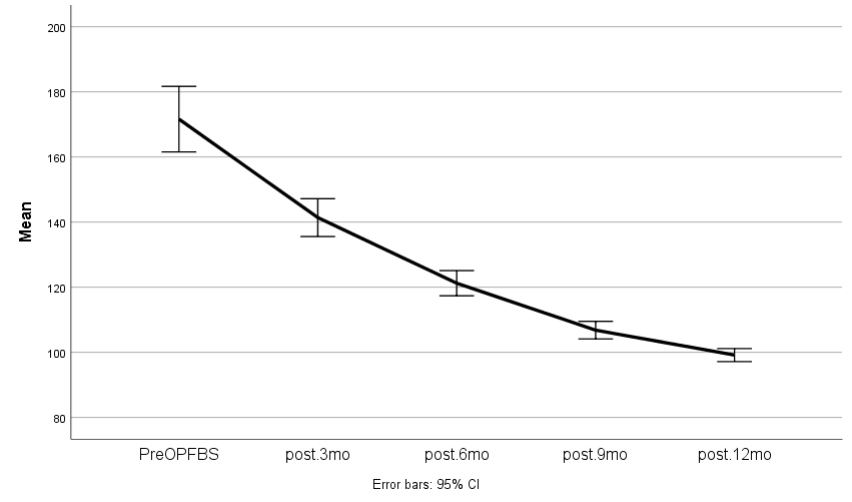
FBS Changes During the Follow-up Period (Pre-Operation, 3 Months, 6 Months, 9 Months, and 12 Months)

**Figure 2:**
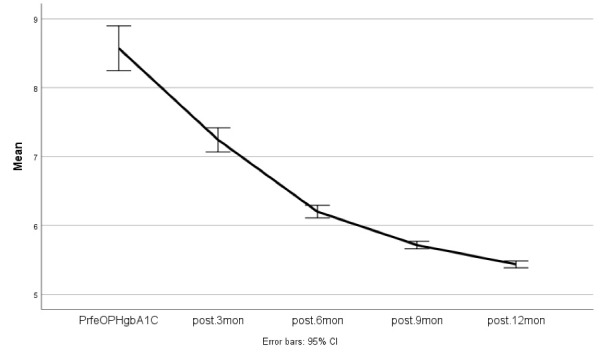
HbA1c Changes During the Follow-up Period (Pre-operation, 3 Months, 6 Months, 9 Months, and 12 Months)

Figure 1 and Figure 2 demonstrate FBS and HbA1c changes during the follow-up period.

## Discussion

 The SASI bypass included both restrictive and malabsortive procedures with few complications in our study. Gastric cancer has moved from the second to the third most common global cancer; however, it has a remarkable prevalence rate in Iran.^14^ This surgery was done among diabetic patients who may be in a need for further evaluations or procedures by endoscopy for their comorbidities like gastric cancer.

 Vertical sleeve gastrectomy, Roux-en-Y gastric bypass, and the mini gastric bypass are the most prevalent bariatric surgeries worldwide^15^ Apart from preserving digestive (e.g., gastric, pyloric, and duodenal) functions, the perfect operation should regulate the neuroendocrine control of both hunger and satiety. Based on this goal, surgeons use digestive adaptation as a surgical technique used for obesity.^9^ Santoro et al in 2012 proposed a technical modification in the sleeve gastrectomy with transit bipartition, known as SASI bypass.^8^

 In Emile and colleagues’ study, SASI bypass resulted in significantly greater improvement in both T2DM and GERD compared to SG (95.8% vs 70% and 85.7% vs 18.2%, respectively). In addition, the reductions in body weight and BMI 12 months after the SASI bypass were significantly greater than those of SG. SASI bypass may be an additional mechanism that contributes to more effective and sustained weight loss compared to SG. SASI bypass resulted in significant improvement of GERD symptoms. Notably, the operation time of SASI bypass was about 15 minutes longer than SG.^10^

 Sewefy et al showed that SASI bypass is associated with some improvements in other obesity-associated comorbidities such as an 89% remission in hypertensive patients.^12^

 Mahdy et al in their study concluded that SASI bypass is an efficient and safe operation for obese patients with type II diabetes, and percentage excess weight loss (calculated from an ideal BMI of 25 kg/m^2^) showed significant improvement; 75% at 6th month and 90% at the first year of follow-up.^9^ Our study showed 60.99% EWL one year after the surgery and 30.39% ± 6.52% TWL% in 59 patients.

 In Salama and colleagues’ study, the mean BMI of the patients decreased from 43.2 kg/m^2^ to 36.4 kg/m^2^ six months after the surgery and reached 29.1 kg/m^2^ after the first year of the surgery.^16^ In the study by Kermansaravi et al, the mean BMI decreased from 44.2 ± 4.3 to 35.5 ± 4.5 after one year.^17^ In our study, BMI decreased from 43.70 ± 5.81 kg/m^2^ to 30.05 ± 4.11 kg/m^2^ after one year.

 In Salama and colleagues’ study conducted on 45 morbidly obese patients, 95% of the patients discontinued multivitamins six months post-surgery, and BMI decreased from 43.2 kg/m^2^ to 29.1 kg/m^2^ in one year. In addition, there was a significant decrease in the plasma levels of insulin, triglyceride, LDL, and fasting blood glucose.^16^

 In conclusion,SASI bypass is a new procedure performed in bariatric surgery that uses restrictive mechanism by sleeve and partial duodenal and small intestine bypass at the same time. This made no significant change in the normal food path from the stomach to the small intestine and no blind sections existed in the GI tract on further evaluations by endoscopy. It is an effective and safe method used for weight loss and controlling type II diabetes mellitus. We should consider the exact intestinal measurement in obese patients as well as procedure selection, because if the common limb becomes short, the complications resulting from the surgery will be high. Therefore, we should follow these patients for longer periods to identify if the surgery is a safe and good enough method for controlling type II diabetes over longer durations. All patients in whom the diabetes was not completely controlled, had to take fewer medications for controlling their disease in comparison to the pre-surgical period.

## References

[1] Chooi YC, Ding C, Magkos F (2019). The epidemiology of obesity. Metabolism.

[2] Colquitt JL, Pickett K, Loveman E, Frampton GK (2014). Surgery for weight loss in adults. Cochrane Database Syst Rev.

[3] Hussain A, Mahawar K, Xia Z, Yang W, El-Hasani S (2020). Obesity and mortality of COVID-19 Meta-analysis. Obes Res Clin Pract.

[4] Pandey A, Chawla S, Guchhait P (2015). Type-2 diabetes: current understanding and future perspectives. IUBMB Life.

[5] Emile SH, Elfeki H. Nutritional deficiency after sleeve gastrectomy: a comprehensive literature review. EMJ Gastroenterol 2017;6(1):99-105 10.33590/emjgastroenterol/10313202.

[6] Kang JH, Le QA (2017). Effectiveness of bariatric surgical procedures: a systematic review and network meta-analysis of randomized controlled trials. Medicine (Baltimore).

[7] Chakravartty S, Tassinari D, Salerno A, Giorgakis E, Rubino F (2015). What is the mechanism behind weight loss maintenance with gastric bypass?. Curr Obes Rep.

[8] Transit Bipartition. Annals of Surgery. 2012 Jul; 256(1), 104–110. 10.1097/sla.0b013e31825370c0. 22609843

[9] Mahdy T, Al Wahedi A, Schou C (2016). Efficacy of single anastomosis sleeve ileal (SASI) bypass for type-2 diabetic morbid obese patients: gastric bipartition, a novel metabolic surgery procedure: a retrospective cohort study. Int J Surg.

[10] Emile SH, Madyan A, Mahdy T, Elshobaky A, Elbanna HG, Abdel-Razik MA (2021). Single anastomosis sleeve ileal (SASI) bypass versus sleeve gastrectomy: a case-matched multicenter study. Surg Endosc.

[11] Safaan T, Bashah M, El Ansari W, Karam M (2017). Histopathological changes in laparoscopic sleeve gastrectomy specimens: prevalence, risk factors, and value of routine histopathologic examination. Obes Surg.

[12] Sewefy AM, Saleh A (2021). The outcomes of single anastomosis sleeve jejunal bypass as a treatment for morbid obesity (Two-year follow-up). Surg Endosc.

[13] Brethauer SA, Kim J, el Chaar M, Papasavas P, Eisenberg D, Rogers A (2015). Standardized outcomes reporting in metabolic and bariatric surgery. Surg Obes Relat Dis.

[14] Fitzmaurice C, Abate D, Abbasi N, Abbastabar H, Abd-Allah F, Abdel-Rahman O (2019). Global, regional, and national cancer incidence, mortality, years of life lost, years lived with disability, and disability-adjusted life-years for 29 cancer groups, 1990 to 2017: a systematic analysis for the Global Burden of Disease Study. JAMA Oncol.

[15] Wang FG, Yan WM, Yan M, Song MM (2018). Outcomes of Mini vs Roux-en-Y gastric bypass: a meta-analysis and systematic review. Int J Surg.

[16] Salama TMS, Sabry K, Ghamrini YE (2017). Single anastomosis sleeve ileal bypass: new step in the evolution of bariatric surgeries. J Invest Surg.

[17] Kermansaravi M, Kabir A, Pazouki A (2020). 1-Year follow-up of single anastomosis sleeve ileal (SASI) bypass in morbid obese patients: efficacy and concerns. Obes Surg.

